# The role of the myo-inositol for the prevention of the gestational diabetes *mellitus*: systematic review

**DOI:** 10.61622/rbgo/2025rbgo29

**Published:** 2025-09-08

**Authors:** Thamyris Thé de Holanda, José Ananias Vasconcelos, Natália Maria de Vasconcelos Oliveira, Joaquim Luiz de Castro Moreira, Shirley Kelly Bedê Bruno, Maria dos Remédios Pacheco de Sousa, Camila Teixeira Moreira Vasconcelos

**Affiliations:** 1 Hospital Geral de Fortaleza Fortaleza CE Brazil Hospital Geral de Fortaleza, Fortaleza, CE, Brazil.; 2 Universidade Federal do Ceará Fortaleza CE Brazil Universidade Federal do Ceará, Fortaleza, CE, Brazil.

**Keywords:** Diabetes, gestational, Inositol, Pregnant people, Premature birth, Incidence, Overweight, Hypertension, pregnancy induced, Primary prevention

## Abstract

**Objective:**

This review evaluated myo-inositol supplementation's effectiveness in pregnant women at high risk for Gestational Diabetes Mellitus (GDM).

**Sources:**

A systematic search in PubMed/MedLine, Cochrane, and VHL databases was conducted using the following terms: "inositol," "diabetes," "gestational diabetes," and "prevention," with no limits on publication period or language. The reference lists were scanned for additional articles.

**Selection criteria:**

Relevant studies were identified by screening titles, abstracts, and full texts, following inclusion and exclusion criteria and eliminating duplicates. One additional study was added after reviewing references.

**Data collection:**

Guided by the PRISMA Statement, data were extracted using Microsoft Excel. The primary outcome was GDM incidence; secondary outcomes included maternal, birth, neonatal health, and adverse effects.

**Data synthesis:**

Five studies were included. Myo-inositol supplementation significantly decreased the incidence of GDM in all studies. One study noted significant weight loss. Three studies found no reduction in insulin treatment needs with myo-inositol supplementation. One study showed a decrease in macrosomia incidence. No decrease in cesarean delivery rates was verified, though one study noted reduced hypertensive disorders’ incidence with myo-inositol. Four studies evidenced no reduction in premature birth or shoulder dystocia. There was no significant difference in hypoglycemia incidence in three studies. One study showed a decrease in Neonatal Intensive Care Unit admissions with myo-inositol supplementation. One patient reported headaches.

**Conclusion:**

Due to study divergences, no clinical recommendations can be made. However, myo-inositol supplementation appears effective in reducing GDM incidence in at-risk pregnant women.

## Introduction

Alterations on the glycemic metabolism are the metabolic disorders, most common on the pregnancy, about 16% of the life birth, born from mothers that were diagnosed with some type of hyperglycaemia. The Gestational Diabetes Mellitus (GDM) is the form most predominant among the types of dysglycemia that affect pregnant women. Such GDM is defined as intolerance to carbohydrates of varying severity, that appears along the pregnancy, although without fulfilling the diagnostic criteria for Diabetes Mellitus (DM). This fact affects between 3% and 25% of the pregnant women, according to the diagnostic criterion and to the evaluated group.^(1)^ In the world, the prevalence of GDM, among pregnant women, aged 20-49, was 16.7%, in 2021.^(2)^

The number of fertile women diagnosed with DM, along the pregnancy and postpartum, has been increasing progressively, in the last two decades. This is due to the increase in maternal age, population growth, sedentary lifestyle and, mainly, prevalence of obesity.^(3)^

Among the risk factors for GDM, we can highlight advanced maternal age, overweight and obesity, genetic diabetes in first degree relatives, GDM in a previous pregnancy, macrosomia in a previous pregnancy and the presence of conditions associated with insulin resistance (Systemic Hypertension, Polycystic Ovary Syndrome).^(1)^

The newborn of the mother with GDM has risk of macrosomia, shoulder dystocia, neonatal hypoglycemia, respiratory distress, obesity, metabolic syndrome and future diabetes.^(4,5)^ Concerning the mothers, the risks are cesarean delivery, hypertensive disturbs and a higher probability and a higher probability to develop DM later on.^(4)^

The main risk factor for the development of DM and metabolic syndrome in women is GDM during a previous pregnancy. Thus, hyperglycemia during pregnancy and the postpartum period is a relevant problem, due not only to the risks of poor perinatal outcomes, but to the increased prevalence of future obesity and DM.^(3)^

The International Federation of Gynecology and Obstetrics (IFGO) and the guidelines of several countries recommend to perform a 2-hour 75g oral glucose tolerance test (GTT) in all pregnant women, between 24 and 28 weeks of gestation, for diagnosis and control of the disease.^(6)^

Therapeutic approaches for GDM include nutritional control, weight control, physical exercise, blood glucose monitoring and pharmacological therapy, if necessary. Several studies have been carried out on the effectiveness of substances such as myo-inositol in preventing GDM and its complications in recent years.^(7)^

Myo-inositol is an isomer of inositol, present in cereals, corn, vegetables and meat, in addition to being synthesized by the organism, mainly by the liver. Despite having pharmacological effects confirmed, myo-inositol is sold as a dietary supplement in several countries.^(8)^ In Brazil, myo-inositol 2g is found as a powder alimentary supplement in sachets, associated with 200 mg of folic acid, myo-inositol 2g, associated with 600 mg of folic acid, and 2g of inositol, associated with 200 mg of folic acid.

Inositol, usually recognized as safe, is present on the United States Food and Drugs Administration (US FDA) list of compounds, which means that such substance is both free of side effects and safe for use during pregnancy.^(9)^

Recent studies have demonstrated that dietary supplementation with myo-inositol has insulin-sensitizing effects.^(4,10)^ As increased insulin resistance, during pregnancy, is the main pathophysiological mechanism of GDM, it is possible to assume that dietary supplementation with myo-inositol may influence on preventing the onset of GDM and its complications.^(11)^

Four recent meta-analyses, involving 28 RCTs (Randomized Clinical Trials) in all, observed similar results in relation to the impact of myo-inositol on the incidence of GDM and on maternal and fetal outcomes of GDM.^(12–15)^ All of these meta-analyses have shown a reduction in the incidence of GDM and premature births with the use of myo-inositol. One of them also has shown a decrease in the incidence of gestational hypertension in overweight and obese pregnant women.^(15)^ No difference in birth weight, cesarean delivery, macrosomia, shoulder dystocia, neonatal hypoglycemia or neonatal intensive care unit (NICU) admission has been observed with the use of myo-inositol, when compared to placebo, in most of the cases studied.

Due to the importance of this theme, a systematic review of five randomized clinical trials was carried out, in order to evaluate the effectiveness of myo-inositol in preventing GFM and its possible complications (birth weight, fetal macrosomia, cesarean delivery, hypertensive disturbs, premature delivery, shoulder dystocia, neonatal hypoglycemia, admission to NICU and need for insulin treatment).

## Methods

### Study design

Systematic review, organized according to the PRISMA (*Preferred Reporting Items for Systematic Reviews and Meta-analyses*) recommendations.^(16)^

### Search strategy

For the literature search, two independent researchers used the following databases: PubMed, Cochrane and VHL, on October 18, 2022. Search terms employed and combined in each database were: "Inositol", "Diabetes", "Gestational Diabetes" and "Prevention". Further searches were performed through reference lists of relevant articles.

### Study selection

**Figure 1:**
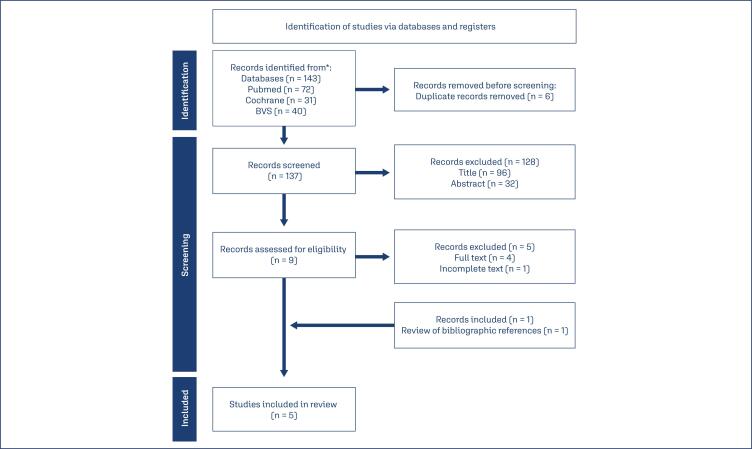
Research Flowchart and study selection

Firstly, the studies were evaluated from their titles. Secondly, the titles that were not excluded, were evaluated from the abstracts and from the whole text, adding the studies according to the criteria of inclusion and exclusion. Duplicated studies were eliminated. After the review of bibliographic references, one study was added, resulting in five selected RCTs, as shown in the flowchart in figure 1.

### Criteria of inclusion

Randomized Clinical Trials that included pregnant women up to 14 weeks of gestation, at high risk for GDM (overweight or obesity and/or Family history of diabetes *mellitus,* confirmed with oral glucose tolerance test, during 24-28 weeks of gestation), with the use of myo-inositol as an intervention, compared to placebo, were included.

### Criteria of exclusion

Multiple pregnancies; pregnant women with type 1 or 2 diabetes *mellitus*; pregnant women undergoing corticosteroid treatment.

### Data extraction

The data were extracted from the selected studies, presenting the first author, the year of publication, the study region, number of participants, participants’ baseline features, intervention protocols, results and reported adverse events.

### Result measures

The primary outcome was the incidence of GDM. Secondary outcomes included maternal health outcomes (hypertensive disease, need for insulin treatment), birth outcomes (preterm birth, cesarean delivery, shoulder dystocia), neonatal health outcomes (birth weight, macrosomia, neonatal hypoglycemia, admission to NICU) and associated adverse effects (Figure 1).

## Results

The main features of each study are summarized in chart 1.

**Chart 1:** Study features

Study	Country	Inclusion criteria	Interventions	Myo-inositol	Control
D’Anna et al. (2013)^(4)^	Italy	(1) GA: 12-13 weeks (2) Pre-gestational BMI < 30 kg/m² (3) FBG < 126 mg/dl, FTG < 200 mg/dl (4) Single pregnancy (5) Caucasian ethnicity (6) 1st degree relative with DM2	Group A: 2g MI + 200mcg FA 2x/day. Group B: 200mcg FA 2x/day.	Group A: N = 99 Age: 31.0 ± 5.3 BMI: 22.8 ± 3.1	Group B: N = 98 Age: 31.6 ± 5.6 BMI: 23.6 ± 3.1
Vitale et al. (2021)^(7)^	Italy	(1) GA: 12-13 weeks (2) Pre-gestational BMI ≥ 25 kg/m², < 30 kg/m² (3) FBG < 126 mg/dl, FTG < 200 mg/dl (4) Singleton pregnancy (5) Caucasian ethnicity	Group A: 2g MI + 200mcg FA 2x/day. Group B: 200mcg FA 2x/day.	Group A N = 110 Age: 27.18 ± 6.03 BMI: 27.00 ± 1.49 FBG: 82.20 ± 12.12	Group B N = 113 Age: 27.95 ± 4.90 BMI: 26.68 ± 1.56 FBG: 83.10 ± 14.10
Santamaria et al. (2016)^(8)^	Italy	(1) GA: 12-13 weeks (2) Pre-gestational BMI ≥ 25 kg/m², < 30 kg/m² (3) FBG < 126 mg/dl, FTG < 200 mg/dl (4) Singleton pregnancy (5) Caucasian ethnicity	Group A: 2g MI + 200mcg FA 2x/day. Group B: 200mcg FA 2x/day.	Group A N = 95 Age: 32.1 ± 4.8 BMI: 26.9 ± 1.3 FBG: 81.09 ± 8.03	Group B N = 102 Age: 32.7 ± 5.3 BMI: 27.1 ± 1.3 FBG: 78.63 ± 6.15
D’Anna et al. (2015)^(10)^	Italy	(1) GA: 12-13 weeks (2) Pre-gestational BMI ≥ 30 kg/m² (3) FBG < 126 mg/dl, FTG < 200 mg/dl (4) Singleton pregnancy (5) Caucasian ethnicity	Group A: 2g MI + 200mcg FA 2x/day. Group B: 200mcg FA 2x/day.	Group A N = 110 Age: 30.9 (18~44) BMI: 33.8 (30.0~46.9) FBG: 83.1 ± 8.5	Group B N = 110 Age: 31.7 (19~43) BMI: 33.8 (30.0~46.0) FBG: 82.3 ± 10.6
Esmaeilzadeh et al. (2023)^(17)^	Iran	(1) GA: 12-14 weeks (2) Pre-gestational BMI ≥ 25 kg/m² < 30 kg/m² (3) FBG < 126 mg/dl, FTG < 200 mg/dl (4) Singleton pregnancy	Group A: 2g MI + 200mcg FA/day. Group B: 400mcg FA/day.	Group A N = 27 Age: 27.8 ± 4.2 BMI: 27.3 ± 1.8 FBG: 84 ± 6.8	Group B N = 29 Age: 29.3 ± 4.4 BMI: 26.9 ± 1.9 FBG: 85.2 ± 6.5

MI - mio-inositol; GA - gestational age; BMI - body mass index; FTG - finger-tip glucose; FBG - fasting blood glucose; FA - folic acid

The five studies included were published between 2013 and 2021, four of which were carried out in Italy and the other in Iran. Sample sizes ranged from 56 to 223. Three studies were carried out in two centers.^(8,10,17)^ The two left were carried out in a single center.

Three studies included non-obese overweight women whose pre-pregnancy body mass index (BMI) was between 25 and 30 kg/m2.^(7,10,17)^ One one study included obese women, whose pre-pregnancy BMI was greater than 30 kg/m2.^(8)^ One study selected women with pre-pregnancy BMI < 30 kg/m2, including non-overweight women.^(4)^ One study included only patients with a family history of DM2.^(4)^

Four studies compared the effect of 4 grams of myo-inositol per day with placebo.^(4,7,8,10)^ Another study compared 2 grams of myo-inositol per day with placebo.^(17)^ The placebo was 200 micrograms of folic acid twice daily or 400 micrograms once daily in all studies. The duration of the intervention in four studies was from the inclusion in this study until delivery.^(4,7,8,10)^ In one study, myo-inositol was used from 14-24 weeks of gestation.^(17)^

In four studies, the diagnosis of GDM was based on the recommendations of the International Association of Study Groups of Diabetes and Pregnancy (IASGDP).^(4,7,8,10)^ One study used the American Diabetes Association (ADA) criteria for diagnosing GDM.^(17)^

All the five studies included evaluated the incidence of GDM. Compared to the control group, in all five studies, myo-inositol supplementation significantly decreased the incidence of GDM. Table 1 presents the incidence of GDM in these five studies, showing a significant reduction in the incidence of GDM in the myo-inositol-treated group.

**Table 1:** Incidence of Gestational Diabetes Mellitus

Results	Author	Myo-inositol n(%)	Control n(%)	p-value
Incidence of DMG	D’Anna et al. (2013)^(4)^	6(6)	15(15.3)	0.04
	Vitale et al. (2021)^(7)^	9(8.2)	24(21.2)	0.006
	Santamaria et al. (2016)^(8)^	11(11.6)	28(27.4)	0.004
	D’Anna et al. (2015)^(10)^	15(14)	36(33.6)	0.001
	Esmaeilzadeh et al. (2023)^(17)^	3(11.1)	11(37.9)	0.038

Three studies evaluated neonatal birth weight.^(4,8,10)^ Only one of them showed significant weight decrease in the myo-inositol, when compared to the control group.^(4)^Table 2 shows the average birth weight in pregnant women who used myo-inositol and those in the control group.

**Table 2:** Secondary results (birth weight)

Result	Author	Myo-inositol m±dp	Control m±dp	p-value
Birth weight (g)	D’Anna et al. (2013)^(4)^	3111 ± 447	3273 ± 504	0.018
	Santamaria et al. (2016)^(8)^	3164.6 ± 462	3221.6 ± 508.2	0.4
	D’Anna et al. (2015)^(10)^	3289 ± 505	3242 ± 579	0.55

Four studies evaluated the incidence of macrosomia. In only one of them, it was verified a decrease of the incidence with the myo-inositol supplementation, when compared to the control group.^(4)^ Four studies evaluated the rate of cesarean deliveries. No decrease was verified with the myo-inositol supplementation in any of the studies.^(4,8,10,17)^ Four studies evaluated the incidence of hypertensive disturbs. In only one of them, it was verified a decrease of the incidence with use of the myo-inositol.^(10)^Table 3 presents secondary results comparing macrosomia incidence and cesarean rates between myo-inositol and control groups.

**Table 3:** Secondary results

Result	Author	Myo-inositol n(%)	Control n(%)	p-value
Macrosomia (>4.000 g)	D’Anna et al. (2013)^(4)^	0 (0)	7 (7.1)	0.007
	Santamaria et al. (2016)^(8)^	1 (1)	5 (4.9)	0.2
	D’Anna et al. (2015)^(10)^	5 (5.1)	5 (4.8)	0.89
	Esmaeilzadeh et al. (2023)^(17)^	2 (7.4)	2 (6.9)	0.941
Caesarean delivery	D’Anna et al. (2013)^(4)^	42.4	43.8	p>0.05
	Santamaria et al. (2016)^(8)^	38 (40)	49 (48)	0.3

Four studies evaluated the incidence of premature birth. When compared to the control group, the myo-inositol supplementation did not reduce the incidence of premature birth.^(4,8,10,17)^ Four studies evaluated the incidence of shoulder dystocia and no difference was verified between the myo-inositol supplementation and the control group.^(4,8,10,17)^ Three studies evaluated the incidence of neonatal hypoglycemia, in which there was no significant difference in the incidence of hypoglycemia between the groups (Table 3).^(4,8,10,17)^

Three studies evaluated the incidence of newborns being admitted to the NICU.^(8,10,17)^ Only one of the studies showed a decrease in the rate of admission to the NICU in the myo-inositol group compared to the control group (Table 3).^(10)^

Four studies evaluated the need to treat patients with insulin. When compared to the control group, the myo-inositol supplementation did not reduce the need for insulin treatment in three studies.^(8,10,17)^ As one of the studies did not present the p-value for the result, it could not be evaluated (Table 3).^(7)^

The five studies evaluated the presence of collateral effects of myo-inositol. Only one patient, who used myo-inositol, reported episodes of headache that, according to the author, had nothing to do with the drug.^(17)^

## Discussion

Several methods have been researched to prevent GDM, such as diet, exercise, insulin, metformin, but their effectiveness has not been proven.^(17)^ Therefore, several studies have been carried out, in order to evaluate the effectiveness of myo-inositol, in prevention of GDM, showing good results and corroborating the result of the present systematic review.^(12–15)^

The present study reveals a decrease in the incidence of GDM with the use of myo-inositol in all RCTs analyzed. Comparing absolute numbers, it was observed a higher decrease in the study carried out by Esmaeilzadeh et al.,^(17)^ in which a 37.9% incidence of GDM in the control group and an 11.1% incidence in the myo-inositol group were verified. There was a higher benefit of myo-inositol in the studies that evaluated overweight/obese pregnant women, when compared to the study that evaluated pregnant women with BMI up to 30 kg/m^2^, revealing a possible benefit of myo-inositol in pregnant women with obesity.

It is known that pregnancy itself is a state of increase of insulin resistance, an effect also caused by overweight and obesity. Together, these effects bring an increased risk of GDM to this population.^(18,19)^ Thus, it would be of great value that a new drug offers the benefit of reducing the risk of GDM, in this group of pregnant women.

One of the studies showed a significant decrease in the birth weight of the newborns.^(4)^ However, recent meta-analyses showed no decrease in weight.^(13,14)^ Another meta-analysis, in turn, shows a decrease in weight only in percentiles.^(11)^ Regarding macrosomia, only one study demonstrated a significant decrease in the incidence of fetal macrossomia.^(4)^ However, three recent meta-analyses present different results.^(11,12,15)^

The article that showed a significant decrease in birth weight, is the same one that shows a decrease in the incidence of fetal macrossomia.^(4)^ The study, presented in such article, focused on women with a family history of DM, not including pregnant women with obesity, and with the lowest mean BMI of all studies (BMI 22.8 ± 3.1). Perhaps we can affirm that myo-inositol has a higher benefit in reducing the weight of newborns of women with lower weight. Thus, there is a need for other studies that include similar population and dose of myo-inositol, in order to evaluate the possible benefits of weight loss and of macrosomia in this specific group.

Evidences also show divergent results regarding the action of myo-inositol on hypertensive disorders. Some of these evidences show benefits.^(15,20)^ In contrast, others do not show.^(11,12)^ In this systematic review, only one study showed a decrease in the incidence of hypertensive disorders, concerning only obese women.^(10)^

Baldassarre et al.^(21)^ suggested that myo-inositol is indispensable in insulin signaling and in improving vascular endothelial function and can be used as an adjuvant treatment in various metabolic diseases, such as endothelial disorders and insulin resistance. A recent systematic review showed a significant reduction in systolic and diastolic blood pressure in non-pregnant patients using Inositol.^(20)^

A meta-analysis, that used RCTs only with women with high BMI, observed a decrease in the incidence of hypertensive disturbs in pregnant women using myo-inositol.^(15)^ This reinforces that the studies that did not showed benefits may be due to small samples. Thus, when analyzing all the data together, in a meta-analysis, the statistical difference can be observed, suggesting that myo-inositol may prevent hypertensive disorders in overweight/obese women.

When analyzing the RCTs in isolation, no benefit of myo-inositol in preventing the incidence of premature birth was observed. However, recent meta-analyses showed a positive impact of the use of myo-inositol in prematurity.^(11–15)^ One study proposed a hypothesis of the effect of myo-inositol in preventing preterm birth, in which the physiological decrease in uteroplacental inositol levels, together with the pro-inflammatory environment of the placenta, causes spontaneous rupture of the placental membrane and the onset of labor birth. Thus, higher levels of inositol in the uterus and placenta, elevated by maternal supplementation, may reduce eicosanoid production, lipid metabolism and secretion of proinflammatory cytokines, which usually affect the uterine placental environment, responsible for the initiation and progression of labor birth, reducing the risk of premature birth.^(22)^

Regarding neonatal hypoglycemia, no single selected study showed a difference in incidence with the use of myo-inositol. Some recent meta-analyses corroborate our results.^(12,15)^ In contrast, others show a decrease in the incidence of neonatal hypoglycemia.^(11,23)^ Such decrease can be explained by the absence of side effects of myo-inositol, in addition to the reduction in cases of GDM with its use, requiring less use of insulin, a drug that can cause neonatal hypoglycemia.^(23)^ Due to such divergences, deeper studies need to be carried out.

Most studies that evaluated insulin treatment showed that there is no difference in the need to use insulin with the use of myo-inositol. Bertrand et al. (2022),^(12)^ in a meta-analysis, agree with the result of this systematic review. Another selected RCT reveals a decrease in the need for insulin in the myo-inositol group. However, as it does not perform statistical analysis, it is not possible to say whether such decrease was significant.^(7)^ Wei et al.^(11)^ and Matarrelli et al. ^(23)^ come to conclusion, in their meta-analyses, that a reduction in the need for insulin use, with myo-inositol, was verified.^(11,23)^ Although Matarrelli et al.^(23)^ did not prove a statistically significant decrease (p=0.053), there was a good reduction in their absolute number.

It was evidenced that the studies, that demonstrated a reduction of neonatal hypoglycemia, coincide with a lower incidence of need for insulin use,^(11,23)^ suggesting a relationship between the use of insulin by the mother and the hypoglycemia, that can occur as an adverse effect in the newborn.^(11,23)^ The divergences among the studies can be attributed to the heterogeneous food intake of pregnant women with GDM, what may lead to a higher or lower need for need for insulin treatment, excluding, thus, partially or completely, any possible benefit of myo-inositol.

As a limitation of this systematic review, we highlight the weakness of evidence to support a clinical recommendation, despite the studies included indicating a statistically significant reduction in the incidence of GDM. Eighty percent of the included RCTs were carried out with women from a single country (Italy), which covers a mostly Caucasian population, restricting the possibility of extending the results to other ethnic groups. In addition, the selected studies evaluate the same main result, but they show secondary results in a heterogeneous way, which makes it difficult to evaluate and to compare them with each other.

The performance of this systematic review aims at improving health outcomes, enabling the implementation of new treatment and diagnostic and supporting protocols related to the prevention of GDM. All these aims taken together show how relevant is this systematic review in obstetrics services.

This review, together with other ones already carried out, shows that, although the current literature shows a reduction in the incidence of GDM in patients using myo-inositol, other secondary outcomes, such as maternal, birth and neonatal outcomes and adverse effects, still show considerable divergence. Thus, more large-scale studies with different populations are needed in order to evaluate the benefit of myo-inositol supplementation on these secondary outcomes, providing, thus, a stronger clinical recommendation to obstetrical services.

## Conclusion

Nutritional supplementation with myo-inositol, whether 2 or 4 grams per day, has been shown to reduce the incidence of GDM in pregnant women at risk of such disease. However, regarding secondary results, there are divergences among the analyzed studies. Therefore, more studies are needed to provide a stronger clinical recommendation regarding the effects of myo-inositol supplementation during pregnancy.

## References

[1] Zajdenverg L, Façanha C, Dualib P, Golbert A, Moisés E, Calderon I (2022). Rastreamento e diagnóstico da hiperglicemia na gestação.

[2] International Diabetes Federation (IDF) (2021). IDF Diabetes Atlas.

[3] Federação Brasileira das Associações de Ginecologia e Obstetrícia, Sociedade Brasileira de Diabetes Organização Pan-Americana da Saúde, Ministério da Saúde (2019). Rastreamento e diagnóstico de diabetes mellitus gestacional no Brasil. Femina.

[4] D’Anna R, Scilipoti A, Giordano D, Caruso C, Cannata ML, Interdonato ML (2013). myo-Inositol supplementation and onset of gestational diabetes mellitus in pregnant women with a family history of type 2 diabetes: a prospective, randomized, placebo-controlled study. Diabetes Care.

[5] Yessoufou A, Moutairou K (2011). Maternal diabetes in pregnancy: early and long-term outcomes on the offspring and the concept of "metabolic memory". Exp Diabetes Res.

[6] Tsakiridis I, Giouleka S, Mamopoulos A, Kourtis A, Athanasiadis A, Filopoulou D (2021). Diagnosis and management of gestational diabetes mellitus: an overview of national and international guidelines. Obstet Gynecol Surv.

[7] Vitale SG, Corrado F, Caruso S, Di Benedetto A, Giunta L, Cianci A (2021). Myo-inositol supplementation to prevent gestational diabetes in overweight non-obese women: bioelectrical impedance analysis, metabolic aspects, obstetric and neonatal outcomes – a randomized and open-label, placebo-controlled clinical trial. Int J Food Sci Nutr.

[8] Santamaria A, Di Benedetto A, Petrella E, Pintaudi B, Corrado F, D’Anna R (2016). Myo-inositol may prevent gestational diabetes onset in overweight women: a randomized, controlled trial. J Matern Fetal Neonatal Med.

[9] Facchinetti F, Appetecchia M, Aragona C, Bevilacqua A, Bezerra Espinola MS, Bizzarri M (2020). Experts’ opinion on inositols in treating polycystic ovary syndrome and non-insulin dependent diabetes mellitus: a further help for human reproduction and beyond. Expert Opin Drug Metab Toxicol.

[10] D’Anna R, Di Benedetto A, Scilipoti A, Santamaria A, Interdonato ML, Petrella E (2015). Myo-inositol supplementation for prevention of gestational diabetes in obese pregnant women. Obstet Gynecol.

[11] Wei J, Yan J, Yang H (2022). Inositol nutritional supplementation for the prevention of gestational diabetes mellitus: a systematic review and meta-analysis of randomized controlled trials. Nutrients.

[12] Bertrand A, Gallot D, Pereira B, Delabaere A (2022). Myoinositol supplementation for the prevention of gestational diabetes in at-risk patients. Systematic review and meta-analysis. Curr Res Pharmacol Drug Discov.

[13] Li L, Fang J (2022). Myo-inositol supplementation for the prevention of gestational diabetes: a meta-analysis of randomized controlled trials. Eur J Obstet Gynecol Reprod Biol.

[14] Liu Q, Liu Z (2022). The efficacy of myo-inositol supplementation to reduce the incidence of gestational diabetes: a meta-analysis. Gynecol Endocrinol.

[15] Mashayekh-Amiri S, Mohammad-Alizadeh-Charandabi S, Abdolalipour S, Mirghafourvand M (2022). Myo-inositol supplementation for prevention of gestational diabetes mellitus in overweight and obese pregnant women: a systematic review and meta-analysis. Diabetol Metab Syndr.

[16] Liberati A, Altman DG, Tetzlaff J, Mulrow C, Gøtzsche PC, Ioannidis JP (2009). The PRISMA statement for reporting systematic reviews and meta-analyses of studies that evaluate health care interventions: explanation and elaboration. PLoS Med.

[17] Esmaeilzadeh S, Ghadimi R, Mashayekh-Amiri S, Delavar MA, Basirat Z (2023). The effect of myo-inositol supplementation on the prevention of gestational diabetes in overweight pregnant women: a randomized, double-blind, controlled trial. Minerva Obstet Gynecol.

[18] Saltiel AR (1990). Second messengers of insulin action. Diabetes Care.

[19] Hadden DR, McLaughlin C (2009). Normal and abnormal maternal metabolism during pregnancy. Semin Fetal Neonatal Med.

[20] Hashemi Tari S, Sohouli MH, Lari A, Fatahi S, Rahideh ST (2021). The effect of inositol supplementation on blood pressure: a systematic review and meta-analysis of randomized-controlled trials. Clin Nutr ESPEN.

[21] Baldassarre MP, Di Tomo P, Centorame G, Pandolfi A, Di Pietro N, Consoli A (2021). Myoinositol reduces inflammation and oxidative stress in human endothelial cells exposed in vivo to chronic hyperglycemia. Nutrients.

[22] Sharma N, Watkins OC, Chu AH, Cutfield W, Godfrey KM, Yong HE (2023). Myo-inositol: a potential prophylaxis against premature onset of labour and preterm birth. Nutr Res Rev.

[23] Matarrelli B, Vitacolonna E, D’Angelo M, Pavone G, Mattei PA, Liberati M (2013). Effect of dietary myo-inositol supplementation in pregnancy on the incidence of maternal gestational diabetes mellitus and fetal outcomes: a randomized controlled trial. J Matern Fetal Neonatal Med.

